# A Comparison of Oral Sensory Effects of Three TRPA1 Agonists in Young Adult Smokers and Non-smokers

**DOI:** 10.3389/fphys.2017.00663

**Published:** 2017-09-07

**Authors:** Eva Ø. Hansen, Lars Arendt-Nielsen, Shellie A. Boudreau

**Affiliations:** Center for Sensory-Motor Interaction (SMI), Department of Health Science and Technology, Faculty of Medicine, Aalborg University Aalborg, Denmark

**Keywords:** transient receptor potential (TRP) channels, oral pain, menthol, nicotine, cinnamaldehyde, vasomotor

## Abstract

This study profiled intra-oral somatosensory and vasomotor responses to three different transient receptor potential (TRP) channels, subfamily A, member 1 (TRPA1) agonists (menthol, nicotine, and cinnamaldehyde) in smoking and non-smoking young adults. Healthy non-smokers (*N* = 30) and otherwise healthy smokers (*N* = 25) participated in a randomized, double-blinded, cross-over study consisting of three experimental sessions in which they received menthol (30 mg), nicotine (4 mg), or cinnamaldehyde (25 mg) chewing gum. Throughout a standardized 10 min chewing regime, burning, cooling, and irritation intensities, and location were recorded. In addition, blood pressure, heart rate and intra-oral temperature were assessed before, during, and after chewing. Basal intra-oral temperature was lower in smokers (35.2°C ± 1.58) as compared to non-smokers (35.9°C ± 1.61) [*F*_(1, 52)_ = 8.5, *P* = 0.005, *post hoc, p* = 0.005]. However, the increase in temperature, heart rate, and blood pressure in response to chewing menthol, nicotine, and cinnamaldehyde gums were similar between smokers and non-smokers. Although smoking status did not influence the intensity of burning, cooling, and irritation, smokers did report nicotine burn more often (92%) than non-smokers (63%) [$\chi_{\left(1,\;\text{N}=55\right)}^{2}$ = 6.208, *P* = 0.013]. Reports of nicotine burn consistently occurred at the back of the throat and cinnamaldehyde burn on the tongue. The cooling sensation of menthol was more widely distributed in the mouth of non-smokers as compared to smokers. Smoking alters thermoregulation, somatosensory, and possibly TRPA1 receptor responsiveness and suggests that accumulated exposure of nicotine by way of cigarette smoke alters oral sensory and vasomotor sensitivity.

## Introduction

Menthol, nicotine, and cinnamaldehyde are only a few of the many naturally occurring sensory stimulants which can be used as surrogate model compounds to activate transient receptor potential (TRP) channels (Namer et al., 2005; Talavera et al., 2009; Earley, 2012) and explore mechanisms contributing to sensory gains and losses. TRP channels are a family of neurally expressed non-selective cation channels (Talavera et al., 2009) that are involved in thermo-, mechano-, and chemo-sensation and play a significant role in acute and chronic (neuropathic) pain and neurogenic inflammation (Calixto et al., 2005; Nilius et al., 2007). Allicin from garlic extract and acroline are found in diesel exhaust are also well-known TRPA1 agonists (for review see Chen and Hackos, 2015). Strategic use of these sensory stimulants enables the study pain mechanisms in humans in order to characterize the associated sensory gains (hyperalgesia, allodynia, and hyperpathia) and sensory loss (hypoalgesia). Hyperalgesia and hypoalgesia to cold or heat stimuli, for example, are clinical symptoms that can reflect an underlying pathology and driving mechanism.

Menthol is an agonist to TRP melastatin 8 (TRPM8) as well as TRP ankyrin 1 (TRPA1) channels (Grace et al., 2014) and is a well-known cooling agent. Although, menthol can evoke cooling sensations, it is commonly used as a surrogate model for cold hypoalgesia (Namer et al., 2005), and is often perceived as painful. Pain and the sensation of burning occur in association with nicotine application to the oral tissues (Hummel et al., 1992; Jensen et al., 2016; Nielsen et al., 2016) or skin (Gore and Chien, 1998). Unlike menthol, the perception of pain and burning are primarily mediated by activation of TRPA1 receptors (Talavera et al., 2009; Kichko et al., 2015). TRPA1 and nicotinic acetylcholine receptors (nAchRs) present on chemosensory vagal C-fibers and Aδ—fiber cough receptors and contribute to the detection of potential (toxic) irritants. Indeed immunohistochemical studies have identified TRPA1 and TRP vanalloid 1 expression in the oropharynx and larynx (Lee et al., 2007; Bessac and Jordt, 2008). A main reason menthol has been incorporated into nicotine replacement products, such as sprays and gums, is to mask nicotine burn and irritation that would otherwise be detected (Rosbrook and Green, 2016).

Our previous studies showed that menthol in combination with nicotine transiently reduced intra-oral nicotine burning and pain (Nielsen et al., 2016). Interestingly, menthol alone evoked burning sensations and pain, albeit to a lower degree than nicotine, in approximately half of the study participants, and further, these participants reported more intense burning and irritation when menthol and nicotine were applied in combination (Nielsen et al., 2016). In a subsequent study, we found a similar result for cinnamaldehyde alone and in combination with nicotine (Jensen et al., 2016).

In contrast to menthol and nicotine, cinnamaldehyde is the most selective TRPA1 agonist, is known to produce burning sensations and pain (Namer et al., 2005) and is the aldehyde that gives cinnamon its taste. Contrary to expectations, our previous study showed cinnamaldehyde in combination with nicotine did not exert a synergistic effect on intra-oral burning or irritation intensities (Jensen et al., 2016). However, individuals responding to cinnamaldehyde as an irritant demonstrated larger areas of nicotine evoked irritation in the throat (Jensen et al., 2016).

Young adults with long-term exposure to nicotine, via cigarette smoking, represent a pain-free and otherwise healthy population to assess the accumulated effects of nicotine on intra-oral somatosensation and vasomotor activity. For example, following a battery of quantitative sensory testing (QST) it has been shown that smokers show reduced sensitivity to cold and warm thermal pain (Yekta et al., 2012). It is well-established that smokers have higher taste thresholds, possibly mediated by a reduction in number of fungiform papillae on the tongue (Khan, 2016), which suggests that sensory end organ function can be altered by cigarette smoking. It is thus fundamentally interesting to assess whether application of TRPA1 agonists to the tongue, such as menthol, nicotine, and cinnamaldehyde evoke similar responses in young adults with long-term exposure to nicotine as compared to healthy controls. Such information may be useful for developing better smoking cessation products (e.g., gum or sprays) as TPRA1 mediates the effects of tobacco products as well as byproducts of chemotherapeutic agents (for review see Fernandes et al., 2012).

The study profiled intra-oral somatosensory, vasomotor, and temperature responses to orally gum-delivered menthol, nicotine, and cinnamaldehyde in smoking and non-smoking young adults. Assessments included the intensity, location and area of evoked burning, cooling, and irritation in response to chewing three different gums and the associated changes in intra-oral temperature, heart rate, and blood pressure. Lastly, participants completed a taste-experience questionnaire to assess taste, and evoked sensations at the end of the chewing regime.

## Materials and methods

### Participants

Thirty healthy non-smokers (16 females, mean age 22.83 years, SD ± 1.80, mean BMI 23.77, SD ± 2.99) and 25 otherwise healthy smokers (12 females, mean age 22.16 years, SD ± 2.08, mean BMI 24.04, SD ± 2.6) participated in a randomized, double-blinded, cross-over study consisting of three experimental sessions. In accord to the National Health Center for Health Statistics (USA) a smoker was defined as a person who has smoked 100 cigarettes in his or her lifetime and who currently smokes cigarettes (mean smoking time 5.35 year, SD ± 2.37) and a non-smoker was defined as a person who had smoked <100 cigarettes throughout his or her lifetime (“NHIS. Adult Tobacco Use Glossary”, 2015). As a follow-up question, participants were asked to report whether they primarily smoked cigarettes with or without menthol.

All participants were pain free and had no history of chronic pain, neurologic, or psychiatric disorders. In addition, the participants did not have any injury, disease, or scar tissue in the oral cavity. Participants refrained from pain-relieving medicine and alcohol for 24 h, caffeine for 4 h, and additional food and beverages for 1 h before sessions. In addition, participants were instructed not to perform moderate to intense exercise 6 h before in order to avoid exercise induced analgesia or fatigue. Further, participants who consumed spicy food more than four times per week were excluded as this may reduce the attitude and perception of oral burning and irritation intensity evoked by the three gums. After receiving both oral and written information, participants gave informed written consent to participate. The ethics committee of Northern Jutland approved the study (case number N-2013-0043) and the study conducted in accord to the Declaration of Helsinki.

### Chewing gum

For the purpose of this study, three gums were specifically designed (Fertin Pharma A/S, Vejle, Denmark). The gums contained menthol (30 mg), nicotine polacrilex (4 mg), or cinnamaldehyde (25 mg) and all gums contained sweeteners. Participants and investigators were not able to differentiate between gums based on their smell and all gums were of comparable size, color (white) and shape similar to our previous studies (Jensen et al., 2016; Nielsen et al., 2016; see Supplemental Figure 1). The doses of menthol, nicotine, and cinnamaldehyde are based on our previous studies (Jensen et al., 2016; Nielsen et al., 2016) and are within the range of commercially available chewing gum and nicotine rehabilitation products. The solubility of nicotine is much higher than cinnamaldehyde and menthol, however the polacrilex gum technology allows for a more slow release of nicotine from the gum resin.

### Study overview

**Figure 1:**
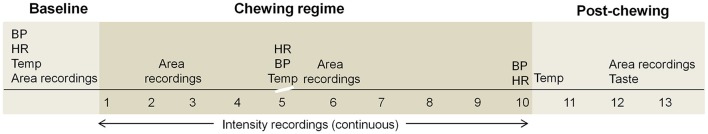
An overview of the experimental session shows all measures prior to, during and following the standardized 10 min chewing regime. A short (15–20 s) interruption in chewing is indicated by the scale break. BP, blood pressure; HR, heart rate; Temp, intra-oral temperature; Taste, taste experience questionnaire.

Participants rested in a seated position, for a minimum of 5 min, prior to onset of the experimental session (see Figure 1). In each experimental session, as separated by a minimum of 24 h, participants received a menthol, nicotine, or cinnamaldehyde gum. Participants chewed the gum at a rate of 40 chews/min, as guided by a metronome, for 10 min. Blood pressure (BP), heart rate (HR), and intra-oral temperature, as captured by a single thermograph image were assessed at baseline, 5 min and following the chewing regime. Recordings of subjective assessments of burning, cooling and irritation intensity occurred throughout the chewing regime on three separate digital visual analog scales (VAS). Drawings of intra-oral burning, cooling and irritation area, on three separate intra-oral body charts, occurred at baseline, 2.5 min, 5 min, and immediately following the chewing regime. Lastly, participants completed a taste-experience questionnaire to assess taste and evoked sensations at the end of the chewing regime.

### Chewing regime

As described in our previous studies, participants performed a 10 min standardized chewing regime consisting of 40 chews/min. Chewing side was altered every 30 s to ensure equal exposure to gum constituents, and swallowing occurred between changes in chewing side. The chewing regime was interrupted only once for ~15–20 s in order assess temperature, as described below.

#### Blood pressure and heart rate

TRPA1 channels are known to alter vascular resistance and subsequently blood flow resulting arterial vasodilation (Pozsgai et al., 2010; Earley, 2012), and nicotine increases HR and BP in response to systemic nicotine absorption (Koch et al., 1980; Fattinger et al., 1997). Different systemic responses, between the smoking and non-smoking populations during and following the chewing of the three gums were assessed by measures of BP and HR using an electronic sphygmomanometer (Omron, USA) at baseline, 5, and 10 min of chewing. The sphygmomanometer cuff was placed around the left upper arm and two BP and HR measurements were performed in a seated position at each time interval. The mean of the two BP and HR assessments was calculated offline and used for further statistical analysis.

#### Intra-oral temperature

Given that TRPA1 agonists are known to elicit vasodilation of the intra-oral tissues (Earley, 2012; Earley and Brayden, 2015) thermography was used as tool to quickly capture the temperature associated changes that can occur with vasodilation. The benefit of using thermography is that it enables a relatively uninterrupted assessment of intra-oral temperature mid-way chewing as a single thermograph takes ~15 s to acquire. Our pre-experimental findings and our earlier studies show that extended interruptions, <1 min can reduce the intensity of the sensory evoked sensations. A thermography camera (SATIR, China) with detector resolution of 384 × 288 was placed 80 cm directly in front of the participants to maximize the camera resolution. The gum was removed from the oral cavity for each thermograph. Participants opened their mouth, as wide as possible, covered their teeth with their lips, and placed the anterior aspect of their tongue-tip behind the lower central incisors. For each thermograph, the corners of the mouth, and the upper and lower lips were marked using the software application SAT report (FLIR, USA) (see Supplemental Figure 2) and the mean temperature of the mouth automatically extracted and recorded offline for further analyses.

#### Burning, cooling, and irritation intensity

The intensity of burning, cooling, and irritation was recorded using a VAS App (Aalborg University, Google Play Store) as displayed on a Samsung Galaxy Note 10.1 tablet. The 10 cm (0–100) VAS bars were anchored with no burning or cooling or irritation and worst imaginable burning or cooling or irritation sensation, respectively. Intensity recordings, sampled at 1 Hz, throughout the 10 min chewing regime were averaged over 30 s intervals. The area under the curve (AUC) and peak intensity for each participant was determined and used for further analyses. Menthol, nicotine and cinnamaldehyde are known to evoke burning sensations (Jensen et al., 2016; Nielsen et al., 2016) and thus correlations between the peak burning intensities were specifically investigated to determine whether those responding more strongly to either menthol or cinnamaldehyde responded more strongly to nicotine.

#### Area and location of intra-oral burning, cooling, and irritation

**Figure 2:**
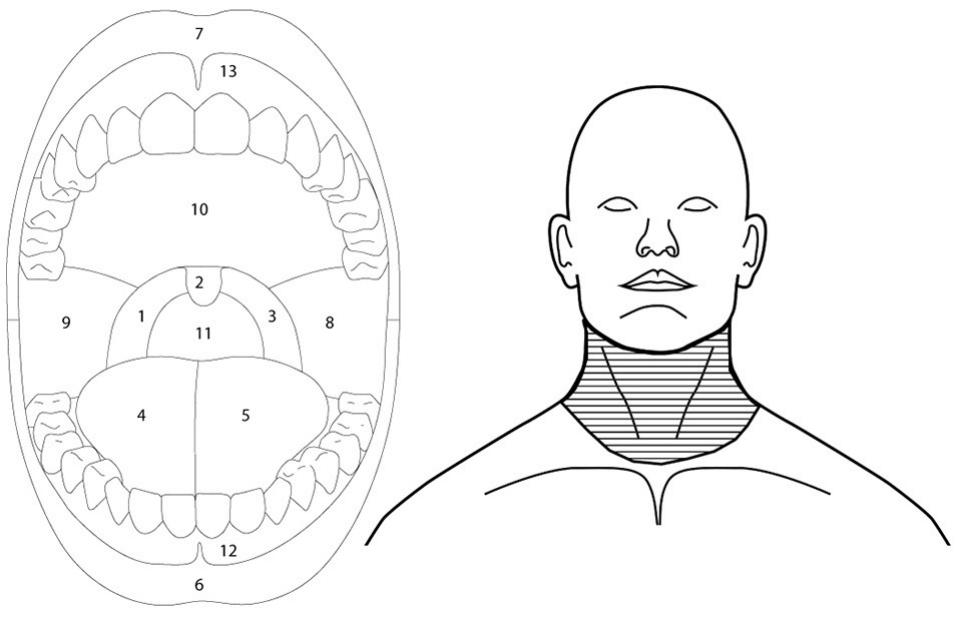
Outlines of the intra-oral (left) and orofacial (right) image used to extract regional somatosensory effects of menthol, nicotine, and cinnamaldehyde. Back of the throat was defined as regions 1, 2, 3, and 11, tongue as regions 4 and 5, and remaining part of the mouth as regions 8, 9, 10, 12, and 13. Lower throat region of the orofacial drawing is indicated by the horizontal line fill.

Participants were asked to draw the area of burning, cooling and irritation, if present, on an intra-oral and orofacial body charts as displayed on a handheld computer tablet (Samsung Galaxy Note 10.) using the Navigate Pain app (Aglance Solutions, Denmark). The intra-oral body chart clearly displayed the tongue, teeth, lips, hard and soft palate, uvula, and oropharynx. The orofacial body chart displayed the neck and face with a closed mouth. Participants used the orofacial body chart to indicate burning, cooling, or irritation sensations in the lower throat. The Navigate Pain App automatically extracted the area, expressed as pixels, for each body chart. The frequency of burning, cooling, and irritation on the tongue, back of the throat, remaining part of the mouth, and lower throat was recorded offline. These locations delineate the most common regions associated with nicotine burn (throat), see Figure 2, and were further subdivided according to anatomy and our previous studies which suggested that cinnamaldehyde may primarily affect the tongue region (Jensen et al., 2016).

#### Taste experience

The taste and sensory experience of the gums was evaluated in a questionnaire following completion of the chewing regime (Jensen et al., 2016; Nielsen et al., 2016). Briefly, participants rated 10 different taste or sensory descriptors on 10 cm VAS bars, as displayed on paper. The VAS bars anchored with 0 as “not at all” and 10 as “to a great extent.” The evaluations of acidity, bitterness, saltiness, sweetness, strength of the taste, burning, warming, cooling, and freshness were used to determine if menthol, nicotine or cinnamaldehyde were more or less dominant.

### Statistical analysis

Statistical analyses were made using IBM SPSS Statistic version 23.0 (SPSS Inc., Chicago, Illonois, USA) and all data were assessed for normal distribution using Q–Q plots.

BP, HR, intra-oral temperature, and taste experience data, were analyzed using a three-way repeated measure (RM) analysis of variance (ANOVA) with gum type, time, or sensory parameters as within and smoking status as a between factor. Bonferroni correction for *post hoc* analysis and two-tailed analysis was used with a significance level set at *P* < 0.05.

Differences between the groups (smokers and non-smokers) in AUC and peak burning, cooling and irritation of the response profiles and the area of burning, cooling and irritation were analyzed using the Mann-Whitney U test. Differences in responses between gum types were analyzed using the Friedman test followed by Wilcoxon signed-rank test as *post hoc* test. *Post hoc* analysis was corrected for multiple comparisons using a Bonferroni correction.

Correlation analyses between peak burning, cooling, and irritation intensity were performed for smokers and non-smokers by determining the Pearson product-moment correlation coefficient. Additionally, correlation analyses were performed to determine whether smoking duration was related to intra-oral temperature and peak burning ratings in response to nicotine.

Comparisons of burning, cooling, and irritation location (tongue, back of the throat, remaining part of the mouth, and lower throat region) between menthol, nicotine, and cinnamaldehyde were performed using Chi square test. When relevant, the Chi square test was used as *post hoc* test.

## Results

Out of the 25 smokers we confirmed only three participants primarily smoked mentholated cigarettes whilst, 17 participants did not. Five participants never responded to the follow-up question regarding menthol preference in cigarettes.

### BP and HR

There were no differences in HR and systolic and diastolic BP between smokers and non-smokers for all gums. All gums elevated HR [*F*_(2, 106)_ = 47.94, *P* < 0.001], systolic BP [*F*_(2, 106)_ = 12.46, *P* < 0.001], and diastolic BP [*F*_(2, 106)_ = 22.18, *P* < 0.001] but this differed across chewing time for HR [*F*_(4, 212)_ = 11.78, *P* < 0.001], systolic BP [*F*_(4, 212)_ = 4.85, *P* = 0.001], and diastolic BP [*F*_(4, 212)_ = 10.23, *P* < 0.001].

HR increased (78.62 ± 13.88 bpm) for all gums at 5 min (*post hoc, p* < 0.001), however only nicotine induced further increases at 10 min (*post hoc, p* < 0.001, see Supplemental Figure 3). Systolic BP increased at 5 min (*post hoc, p* < 0.001) but remained elevated at 10 min (*post hoc, p* < 0.001) for nicotine. For menthol, systolic BP increased at 5 min (*post hoc, p* = 0.021) and showed a tendency to return to baseline at 10 min (*post hoc, p* = 0.07). Systolic BP slightly decrease between 5 and 10 min for cinnamaldehyde (*post hoc, p* = 0.036). Diastolic BP increased for menthol, nicotine and cinnamaldehyde at 5 min (mean increase: 76.04 ± 7.40 mmHg, *post hoc, p* < 0.001) however only menthol and cinnamaldehyde returned to baseline as indicated by a reduction in BP from 5 min to 10 min (*post hoc, p* < 0.002).

### Intra-oral temperature

**Figure 3:**
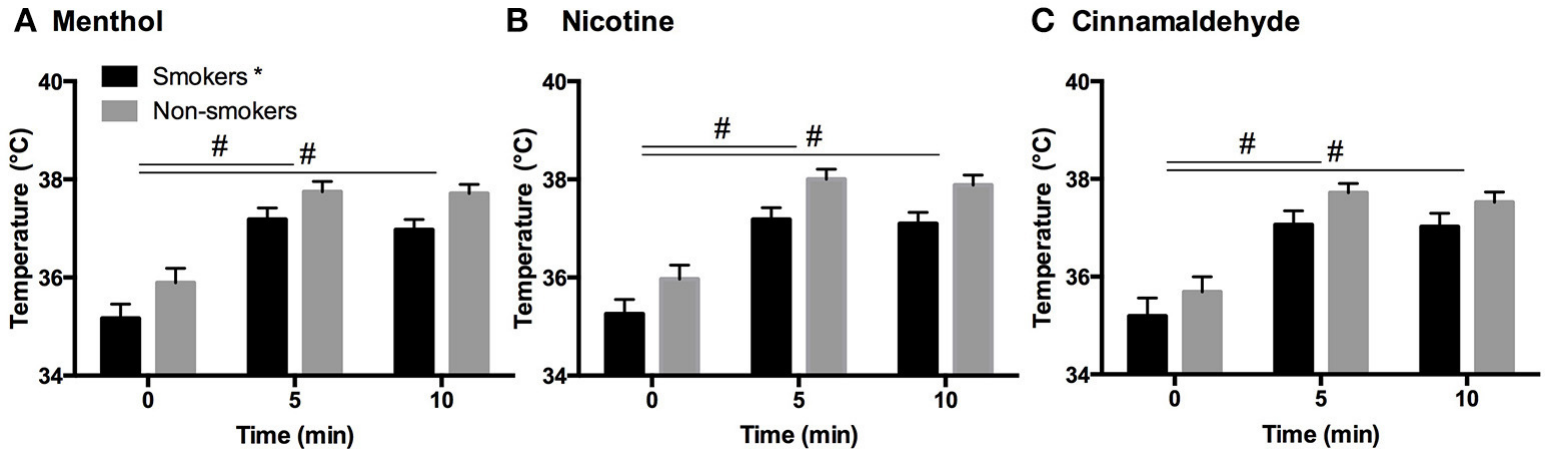
Intra-oral temperature in response to menthol **(A)**, nicotine **(B)**, and cinnamaldehyde **(C)** before, during (5 min) and immediately after chewing (10 min). The basal temperature for smokers was shown to be lower compared to non-smokers, however, an elevation in temperature in the first half of chewing was evident for both smokers and non-smokers and in response to all gums. ^*^Significant difference between groups. ^#^Significant change in temperature over time. Data presented as mean ± SEM.

Generally, smokers demonstrated lower basal intra-oral temperatures (35.2°C ± 1.58) as compared to non-smokers (35.9°C ± 1.61) [*F*_(1, 52)_ = 8.5, *P* = 0.005, *post hoc, p* = 0.005] see Figure 3. However, basal intra-oral temperatures were not correlated to smoking duration (*r* = −0.14, *P* = 0.5). In response to all gums, smokers and non-smokers showed a similar increase in intra-oral temperature at 5 min [*F*_(2, 83)_ = 352.3, *P* = 0.001, *post hoc, p* < 0.001] and remained elevated thereafter (*post hoc, p* < 0.001).

### Intensity of burning, cooling, and irritation

**Figure 4:**
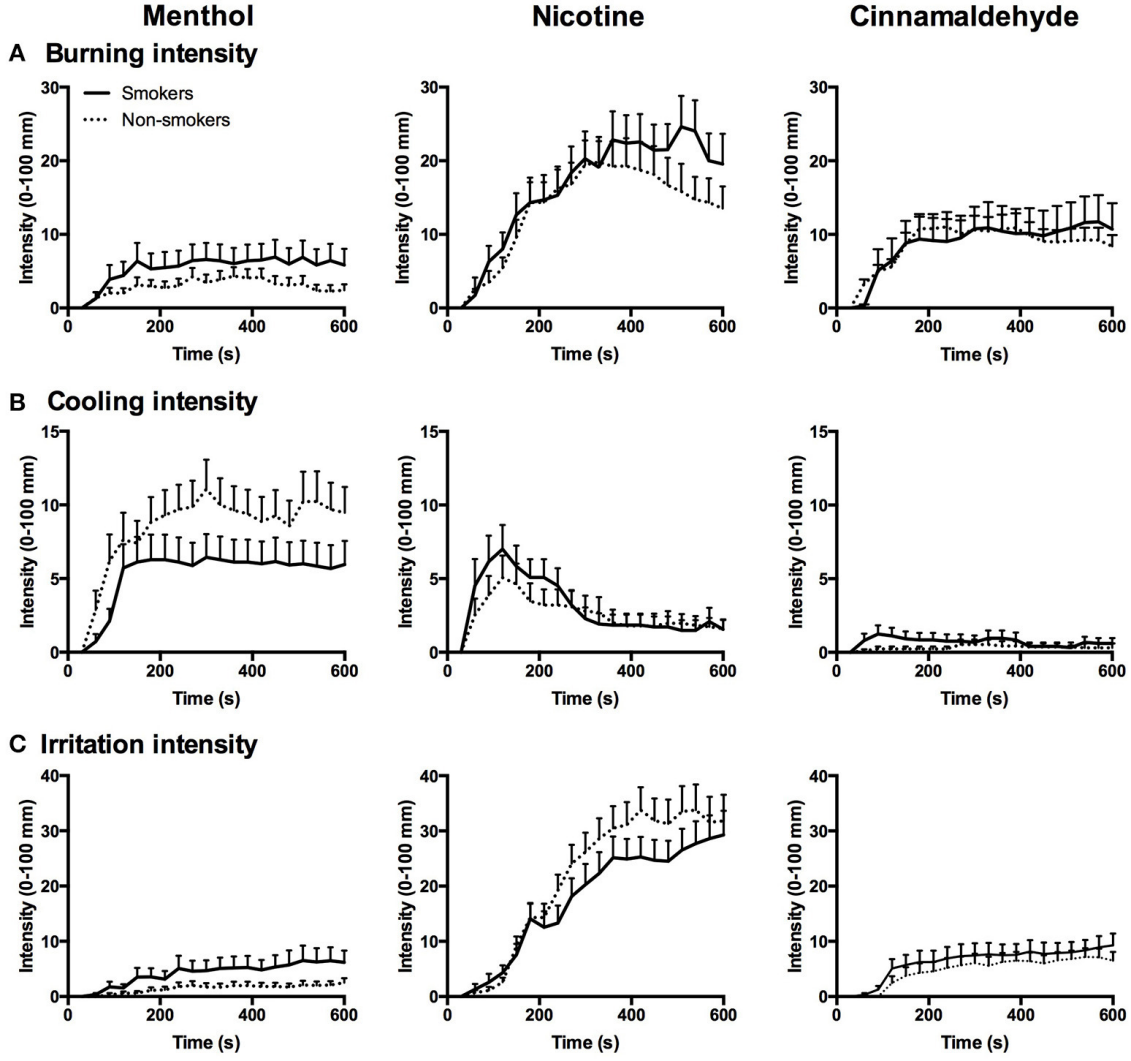
Burning **(A)**, cooling **(B)**, and irritation **(C)** intensity in response to menthol, nicotine, and cinnamaldehyde for smokers and non-smokers, data presented as mean ± SEM for every 30 s interval over the 10 min chewing regime.

The intensity of the burning, cooling, and irritation, as reflecting in the AUC, did not differ between smokers and non-smokers (*P* > 0.162) but differed between gums for burning [$X_{\left(2\right)}^{2}$ = 31.64, *P* < 0.001], cooling [$X_{\left(2\right)}^{2}$ = 32.98, *P* < 0.001], and irritation [$X_{\left(2\right)}^{2}$ = 63.18, *P* < 0.001] as shown in Figure 4.

AUC of the burning response profile were greater for nicotine as compared to menthol (*post hoc Z* = −4.87, *p* < 0.001) and cinnamaldehyde (*post hoc Z* = −4.43, *p* < 0.001), and cinnamaldehyde was greater than menthol (*post hoc Z* = −2.66, *p* = 0.008). Additionally, AUC of the cooling response profile were greater for menthol as compared to nicotine (*post hoc Z* = −4.18, *p* < 0.001) and cinnamaldehyde (*post hoc Z* = −4.19, *p* < 0.001), and nicotine was greater than cinnamaldehyde (*Z* = −5.14, *p* < 0.001). Lastly, AUC of the irritation response profile were greater for nicotine as compared to menthol (*post hoc Z* = −5.75, *p* < 0.001) and cinnamaldehyde (*post hoc Z* = −5.88, *p* < 0.001), and cinnamaldehyde was greater than menthol (*post hoc Z* = −3.68, *p* < 0.001).

Peak burning, cooling, and irritation responses, as shown in Figure 4, did not differ between smokers and non-smokers (*P* > 0.107) but differed between gums for burning [$X_{\left(2\right)}^{2}$ = 43.09, *P* < 0.001], cooling [$X_{\left(2\right)}^{2}$ = 32.2, *P* < 0.001], and irritation [$X_{\left(2\right)}^{2}$ = 67.52, *P* < 0.001].

Peak burning was greater for nicotine as compared to cinnamaldehyde (*post hoc Z* = −4.14, *p* < 0.001) and menthol (*post hoc Z* = −5.08, *p* < 0.001), and cinnamaldehyde was greater than menthol (*post hoc Z* = −3.43, *p* = 0.001). Additionally, peak cooling was greater for menthol as compared to cinnamaldehyde (*post hoc Z* = −5.10, *p* < 0.001) and nicotine (*post hoc* Z = −2.45, *p* = 0.014), and nicotine also showed greater reports of peak cooling as compared to cinnamaldehyde (*post hoc Z* = −3.93, *p* < 0.001). Lastly, peak irritation responses were greater for nicotine as compared to cinnamaldehyde (*post hoc Z* = −5.78, *p* < 0.001) and menthol (*post hoc Z* = −5.78, *p* < 0.001), and cinnamaldehyde was greater than menthol (*post hoc Z* = −3.62, *p* < 0.001).

**Figure 5:**
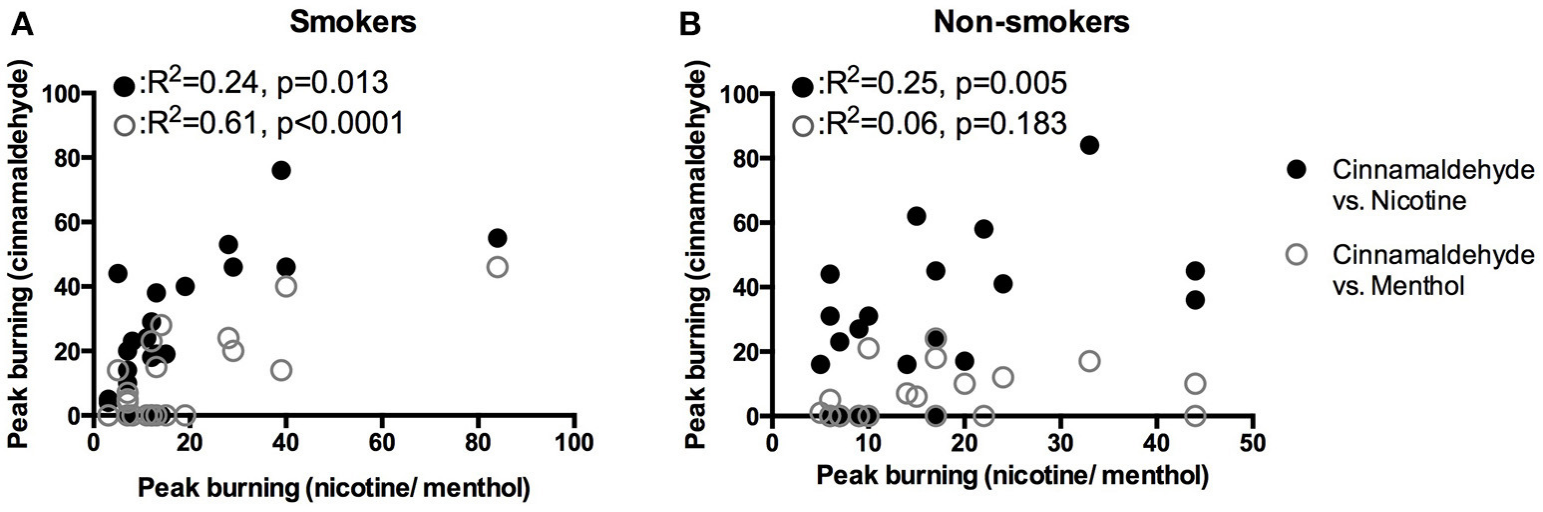
Correlations of peak burning intensity in response to the nicotine, menthol, and cinnamaldehyde for smokers **(A)** and non-smokers **(B)**.

Smoking duration was not correlated to the peak burning intensity (*R* = 0.0, *P* = 0.999). As shown in Figure 5B, peak burning evoked by cinnamaldehyde and nicotine were correlated for non-smokers. Whereas, peak burning evoked by cinnamaldehyde was correlated to nicotine and menthol for smokers (Figure 5A).

### Area of intra-oral burning, cooling, and irritation

#### Mouth

**Figure 6:**
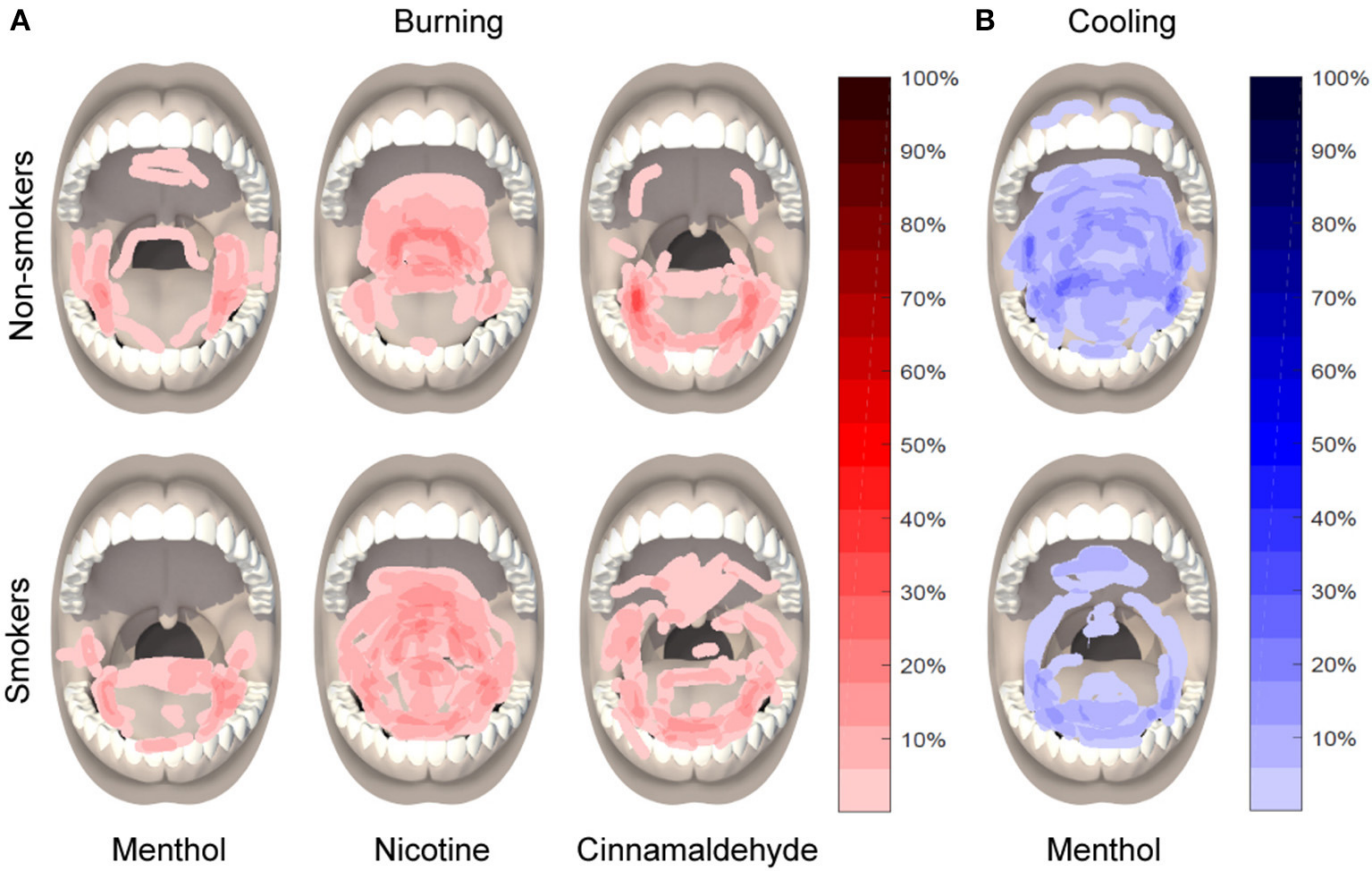
Location and distribution of burning in response to menthol, nicotine, and cinnamaldehyde **(A)** and cooling in response to menthol **(B)**, as assessed 5 min into the 10 min chewing regime.

The area of burning, cooling, or irritation between smokers and non-smokers did not differ for menthol, nicotine, or cinnamaldehyde (*P* > 0.088). However, the area of burning [$X_{\left(2\right)}^{2}$ = 9.89, *P* = 0.007], cooling [$X_{\left(2\right)}^{2}$ = 49.77, *P* < 0.001], and irritation [$X_{\left(2\right)}^{2}$ = 35.41, *P* < 0.001] differed between gum type, as shown in Figure 6.

**Figure 7:**
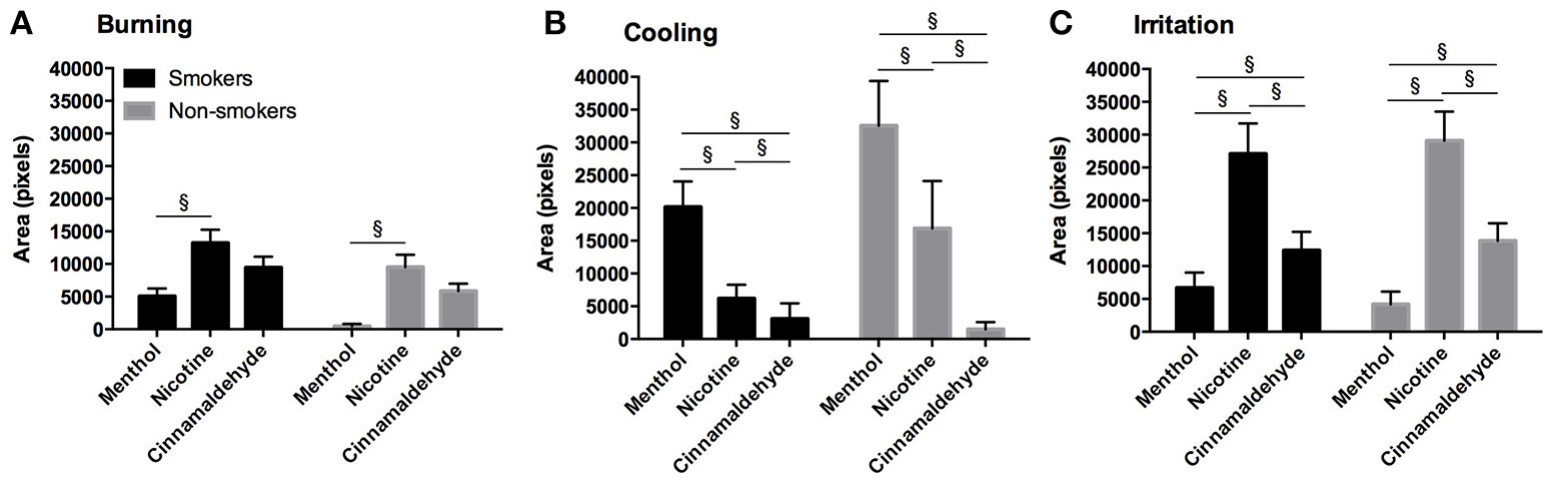
Burning **(A)**, cooling **(B)**, and irritation **(C)** area (as expressed in pixels) for the mouth in response to menthol, nicotine and cinnamaldehyde gums for smokers and non-smokers. The responses were similar for smokers and non-smokers. ^§^Significant difference between gum types. Data presented as mean ± SEM.

The area of burning was greater for nicotine as compared to menthol (*post hoc Z* = −4.53, *p* < 0.001) and a similar tendency was observed between nicotine and cinnamaldehyde (*post hoc Z* = −1.771, *p* = 0.076). The area of cooling was greatest for menthol as compared to nicotine (*post hoc Z* = −3.91, *p* < 0.001) and cinnamaldehyde (*post hoc Z* = −2.97, *p* = 0.003). The area of cooling was also greater for nicotine as compared to cinnamaldehyde (*Z* = −5.28, *p* < 0.001). Lastly, the area of irritation was greater for nicotine and cinnamaldehyde as compared to menthol (*post hoc Z* = −5.79, *p* < 0.001 and *Z* = −3.04, *p* = 0.002; respectively) and nicotine was greater than cinnamaldehyde (*post hoc Z* = −3.89, *p* < 0.001). These results are summarized in Figure 7.

#### Throat

**Figure 8:**
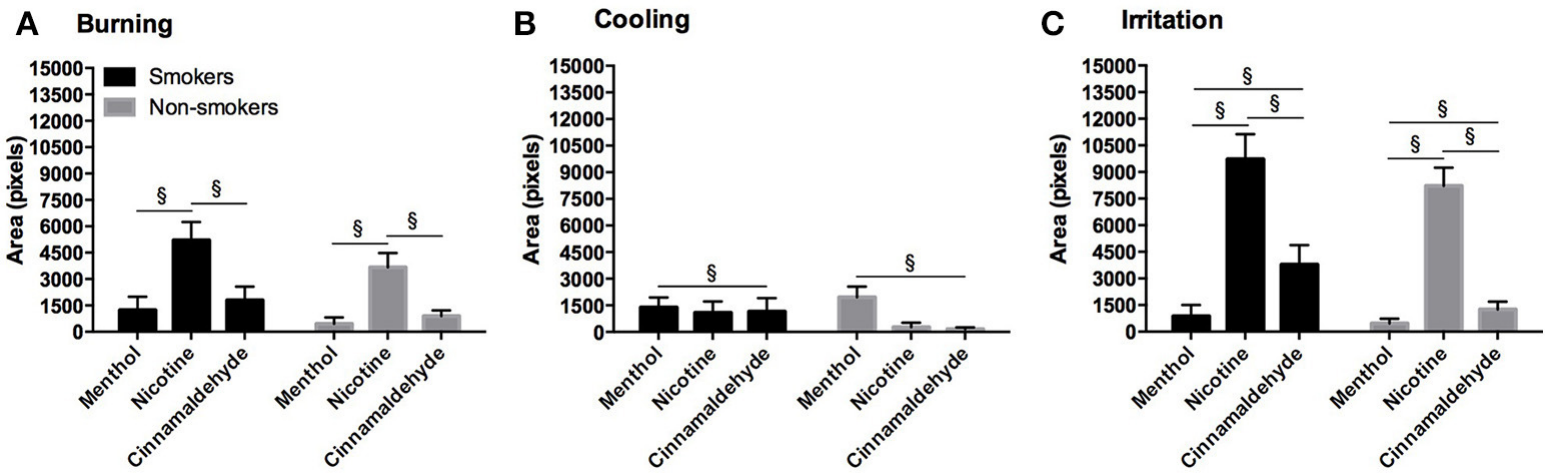
Burning **(A)**, cooling **(B)**, and irritation **(C)** area (as expressed in pixels) for the throat in response to menthol, nicotine and cinnamaldehyde gums for smokers and non-smokers. The responses were similar for smokers and non-smokers. ^§^Significant difference between gum types. Data presented as mean ± SEM.

The total area of burning, cooling, and irritation, as shown in Figure 8, between smokers and non-smokers did not differ for menthol or nicotine and no difference in burning and cooling for cinnamaldehyde was found (*P* > 0.248). However, smokers demonstrated greater irritation responses as compared to non-smokers for cinnamaldehyde (*P* = 0.035).

The area of burning [$X_{\left(2\right)}^{2}$ = 30.91, *P* < 0.001], cooling [$X_{\left(2\right)}^{2}$ = 7.44, *P* = 0.024], and irritation [$X_{\left(2\right)}^{2}$ = 69.35, *P* < 0.001] differed between menthol, cinnamaldehyde, and nicotine. Generally, the area of burning was greatest for nicotine as compared to cinnamaldehyde and menthol (*post hoc Z* = −3.64, *p* < 0.001 and *Z* = −4.73, *p* < 0.001; respectively), whereas, the area of cooling was greater only for menthol as compared to cinnamaldehyde (*post hoc Z* = 2.44, *p* = 0.015). Lastly, the area of irritation was greater for nicotine and cinnamaldehyde as compared to menthol (*post hoc Z* = −6.02, *p* < 0.001 and *Z* = −3.30, *p* = 0.001; respectively) and nicotine was greater than cinnamaldehyde (*post hoc Z* = −5.073, *p* < 0.001).

#### Frequency and location of burning, cooling, and irritation

The frequency of burning reports (occurrences) in response to nicotine, as shown in Table 1, was greater for smokers (92%) than non-smokers [63%, $\chi_{\left(1,\;\text{N}=55\right)}^{2}$ = 6.208, *P* = 0.013] with no additional observed differences for the other gums (*P* > 0.101).

**Table 1:** A summary of the burning sensations reported for smokers and non-smokers in response to the three gums.

	**Menthol**	Nicotine^*^	**Cinnamaldehyde**
Smokers (%)	52	92	84
Non-smokers (%)	47	63	77

* *Significant difference between smokers and non-smokers (P < 0.05)*.

The locations of burning differed between menthol, nicotine and cinnamaldehyde for the back of the throat [$\chi_{\left(2,\;\text{N}=55\right)}^{2}$ = 6.790, *P* = 0.034], the remaining part of the mouth [$\chi_{\left(2,\;\text{N}=55\right)}^{2}$ = 6.746, *P* = 0.034], the lower throat region [$\chi_{\left(2,\;\text{N}=55\right)}^{2}$ = 30.491, *P* < 0.001]. Only a tendency was observed for the tongue [$\chi_{\left(2,\;\text{N}=55\right)}^{2}$ = 5.415, *P* = 0.067]. Nicotine burn occurred more frequently in the lower throat region when compared to cinnamaldehyde and menthol evoked burn (*post hoc p* < 0.001), however cinnamaldehyde burn did occur more frequently than menthol (*post hoc p* = 0.017). Indeed, nicotine burn occurred more frequently than menthol for the back of the throat and the remaining part of the mouth (*post hoc p* < 0.013).

The location of cooling also differed between menthol, nicotine and cinnamaldehyde for the tongue [$\chi_{\left(2,\;\text{N}=55\right)}^{2}$ = 23.669, *P* < 0.001], the back of the throat [$\chi_{\left(2,\;\text{N}=55\right)}^{2}$ = 43.558, *P* < 0.001], and the remaining part of the mouth [$\chi_{\left(2,\;\text{N}=55\right)}^{2}$ = 46.889, *P* < 0.001] but not the lower throat region [$\chi_{\left(2,\;\text{N}=55\right)}^{2}$ = 5.631, *P* = 0.060]. For the tongue, back of the throat, and the remaining part of the mouth, menthol cooling occurred more frequently as compared to cinnamaldehyde and nicotine (*post hoc p* < 0.003). Interestingly, nicotine cooling occurred more frequently for the tongue, back of the throat, and the remaining part of the mouth as compared to cinnamaldehyde (*post hoc p* < 0.042).

The location of irritation differed between menthol, nicotine, and cinnamaldehyde for the tongue [$\chi_{\left(2,\;\text{N}=55\right)}^{2}$ = 11.136, *P* = 0.004], the back of the throat [$\chi_{\left(2,\;\text{N}=55\right)}^{2}$ = 24.188, *P* < 0.001], the remaining part of the mouth [$\chi_{\left(2,\text{N}=55\right)}^{2}$ = 27.367, *P* < 0.001], and the lower throat region [$\chi_{\left(2,\;\text{N}=55\right)}^{2}$ = 61.257, *P* < 0.001]. For all regions cinnamaldehyde irritation occurred more frequently than menthol (*post hoc p* < 0.017). Nicotine irritation occurred more frequently than cinnamaldehyde and menthol at the back of the throat, the remaining part of the mouth, and the lower throat region (*post hoc p* < 0.013) with the exception for the tongue for which cinnamaldehyde irritation occurred most frequently (*post hoc p* = 0.015).

### Taste experience

Taste experiences, as shown in Supplemental Figure 4, did not differ between smokers and non-smokers but did differ between gums [*F*_(8, 447)_ = 24.15, *P* < 0.001]. Similar to the burning and cooling responses, the taste questionnaire re-confirmed that the intensity of burning was greater for nicotine as compared to cinnamaldehyde (*post hoc p* < 0.001) and both burning and warming was greater for nicotine and cinnamaldehyde as compared to menthol (*post hoc p* < 0.013). Cooling and ratings of perceived freshness were greater for menthol as compared to cinnamaldehyde and nicotine (*post hoc p* < 0.001) and cooling and freshness was greater for nicotine compared to cinnamaldehyde (*post hoc p* < 0.024). Sweetness was greater for menthol and cinnamaldehyde as compared to nicotine (*post hoc p* < 0.001). Strength of taste was greater for cinnamaldehyde as compared to menthol (*post hoc p* < 0.001) and bitterness was greater for nicotine as compared to menthol (*post hoc p* = 0.001).

## Discussion

This study assessed oral sensory, vasomotor, and cardiovascular responses to three TRPA1 agonists (nicotine, cinnamaldehyde, and menthol) applied to the oral tissues in smoking and non-smoking populations. Smoking status was associated with decreased basal intra-oral temperatures increased reports of nicotine burn. The intensity of the oral sensory responses in response to all three TRPA1 agonists correlated for smokers whereas only the intensity of nicotine and cinnamaldehyde burn correlated in non-smokers. In general, the location of burning, cooling, and irritation differed in response to the three TRPA1 agonists. The intensity and frequency of nicotine burn and irritation was evidently greater, with the exception of cinnamaldehyde burn on the tongue.

### Intra-oral temperature and cardiovascular responses

Altered intra-oral basal temperatures in smokers suggest lower homeostatic set points may be a consequence of long-term exposure to cigarette smoke and/or nicotine exposure. However, these otherwise healthy smokers showed a similar temperature increase in response to all three TRPA1 agonists, suggesting an intact thermoregulation response. Our previous studies have shown that chewing a placebo gum containing only sweeteners can lead to a similar increase intra-oral temperature as observed in this present study (Jensen et al., 2016; Nielsen et al., 2016). Additionally, we have shown that associated temperature increases occur in the orofacial regions, such as the forehead, but not in distant locations, such as the hand in response to the 10 min (nicotine) chewing gum regime (Jensen et al., 2016). The changes in intra- and orofacial temperature are not systemically driven. A limitation is that the chewing activity can mask subtle changes in intra-oral temperature that would otherwise emerge, if for example, the TRPA1 agonists were topically to a relatively confined intra-oral region. If nicotine, cinnamaldehyde, and menthol preferentially evoke burning and cooling sensations in select intra-oral regions, then this could also be true for changes in temperature. Future studies incorporating immunohistochemistry could verify distribution differences in TRPA1 expressions in the oral cavity and throat regions.

Cigarette smoking increases heart rate within minutes of the first puff (Karakaya et al., 2007). Nicotine gum technology releases nicotine in slower more controlled fashion in order to reduce the “hit” effect and curb cravings. Therefore, a slower systemic effect, as reflected in HR and BP, may have attenuated any differences between smokers and non-smokers. In accord to our placebo-controlled studies, chewing activity itself leads to an initial and transient increase in HR and BP. The chewing activity would explain the elevations in HR in response to cinnamaldehyde and menthol gums (Nielsen et al., 2016). Additional increases in HR and BP in response to nicotine gum, as measured at the end of the chewing regime (10 min), are attributed to the systemic effect and activation of the sympathetic nervous system by catecholamine release and subsequent vasoconstriction (Benowitz, 1997; Fattinger, Verotta, and Benowitz, 1997; Balaji, 2008). The first post-chewing assessment of HR and BP occurred after 5 min, which limits our knowledge on whether rapid differential changes occurred between non-smokers and smokers upon initial exposure. However, nicotine gum is designed for a gradual and slow release and differences between smoker and non-smokers are more likely emerge following longer chewing regimes, which was not explored in this study. In this study, basal HR or BP between smoking and non-smoking young adults did not differ. A larger study consisting of 133 smokers and 165 non-smokers showed higher resting HR-values for young adult smokers (Papathanasiou et al., 2013), indicating that much larger sample sizes may be required to tease out these differences. Epidemiological investigations show no consistent effect of long-term cigarette smoking on resting BP (Green et al., 1986; Istvan et al., 1999) but a moderate *increase* has been suggested (Istvan et al., 1999). In summary, the similar increases in intra-oral temperature, HR and BP profiles in response to nicotine do not provide any evidence of increased peripheral chemo sensitivity (Najem et al., 2006) in young smokers as compared to non-smokers. The evident reduction in basal intra-oral temperature may underlie the differences in burning, cooling, and irritation profiles observed in this study.

### Burning, cooling, and irritation responses

The psychophysical assessments revealed only a few main differences between smoking and non-smoking young adults in response to the three TRPA1. A long-term and accumulated exposure to nicotine, by way of cigarette smoke, did not alter the intensity of burning, cooling, and irritation sensations. Our finding of decreased basal intra-oral temperatures is in agreement with impaired thermosensation found in smoking populations, as reflected by reduced warm and cold detection thresholds in the lingual region (Yekta et al., 2012) and cold detection thresholds in the tongue (Jensen et al., 2016). The similar intensities of evoked burning, cooling, or irritation in response to the three TRPA1 agonists between smokers and non-smokers appears contradictory. It is possible that basal temperature does not influence the overall sensitivity of intra-oral TRP receptors. Although, such a suggestion would be at odds with previous studies showing very clearly that warming skin surfaces prior to or following application of other TRP agonists, such as capsaicin, can enhance the intensity of pain and irritation (Cavallone et al., 2013; Andersen et al., 2015). The lower intra-oral temperature in smokers could reflect a shift in the thermo-somatosensory response profile in which thermosensitive channels remain functionally intact and respond to the same extent when provoked. A limitation of this study is that we did not enquire about oral behaviors following smoking, such as the use of mints or mouthwash. Conceivably these behaviors could influence the perceived differences in oral sensory responses.

For smokers, peak nicotine as well as menthol burn correlated to cinnamaldehyde burn. Further, the area of irritation evoked by cinnamaldehyde was larger for smokers as compared to non-smokers. The additional correlation in smokers suggests that smoking may reduce the specificity of the response profile of TRPA1 agonists. The reduced specificity would explain why smokers more often reported burning and cooling by menthol, nicotine, and cinnamaldehyde in response to the three TRPA1 agonists.

### Differential intra-oral location effects

Unlike non-smokers, almost all smokers reported burning in response to nicotine and this typically occurred in the back of the throat. In smokers, the occurrence of burning appeared to affect a wider distribution of intra-oral regions, as revealed by the oral pain drawings, including the back of the throat as well as the tongue. However, a method to assess the extent of location distribution has yet to be developed. Only smokers reported irritation of the lower throat region in response to cinnamaldehyde. More often, non-smokers reported nicotine burn in the back of the throat and lower throat region and cinnamaldehyde burn on the tongue. Menthol cooling tended to occur more often in the mouth than throat. However, nicotine replacement therapy (NRT) products combine menthol to reduce burning and irritation in the throat. The location of perceived cooling may contribute to the effectiveness of menthol in smoking individuals.

For most intra-oral areas nicotine irritation was larger than cinnamaldehyde, yet the opposite occurred for the tongue. This finding is consistent with our previous study. Nicotine and cinnamaldehyde differ in their affinity for TRPA1, as cinnamaldehyde is an electrophilic agent known to activate TRPA1 by covalent modification of cysteine residues on the channel, and nicotine is a non-electrophilic agent known to activate TRPA1 by non-covalent interactions (Talavera et al., 2009). It is unclear whether the mechanisms by which these substances bind with TRPA1 would explain these results. However, nicotine overpowered cinnamaldehyde (Jensen et al., 2016) and menthol (Nielsen et al., 2016) evoked sensations when applied in combination. Nonetheless, application of topical agents by way of creams or intra-oral sprays should consider location, as there appears to be intra-oral regions more and less affected by menthol, nicotine and cinnamaldehyde.

### Effects of menthol, nicotine, and cinnamaldehyde

Menthol, nicotine, and cinnamaldehyde evoked differing intensities of burning (nicotine > cinnamaldehyde > menthol) and this may be due to dose and solubility properties or parameters of the substances. To date it is unclear how to match the dose of each substance a priori to induce the same perceived intensity of burning, cooling, and irritation. Future studies could employ a dose-response design to estimate these parameters. A cooling response was expected by menthol, yet this study confirmed that nicotine and cinnamaldehyde can evoke cooling as well. Only nicotine and menthol gums produced comparable cooling sensations and supports that TRPA1 does contributes to cooling sensations. Menthol is additive to reduce the burning sensation in NRT products. In this present study nearly two-thirds of those assessed reported cooling responses to menthol, however half reported burning responses. Rosbrook and Green (2016) investigated the impact of menthol on burning sensations when combined with nicotine at low and high concentrations in acute smokers. A low concentration of nicotine (0.5%) combined with menthol resulted in enhanced nicotine burn. In contrast, high concentration (3.5%) of nicotine combined with menthol attenuated nicotine burn.

This present study reconfirmed that 30 mg of menthol produced notable burning and cooling effects. Our previous study showed 4 mg of nicotine combined with 30 mg of menthol attenuates nicotine burn but only for those not responding to menthol as an irritant (Nielsen et al., 2016). Although not contradictory to the bi-directional effects of low and high concentrations of menthol, our previous findings suggest that menthol may not reliably produce a “masking” effect in all individuals. Individuals reporting burning responses to menthol alone demonstrated an enhancement of nicotine burn; especially in the throat (Nielsen et al., 2016). The menthol dose used in this present study is comparable to commercial NRT products and these products combine menthol with nicotine to attenuate nicotine burn. The significance of menthol burn is that those responding to menthol and/or cinnamaldehyde as an irritant are likely to respond more strongly to nicotine alone or in combination.

## Conclusion

The dynamic response to TRPA1 activation by way of nicotine, cinnamaldehyde, and menthol gum appears to be intact in young healthy adult smokers. Healthy adult smokers present with lower basal intra-oral temperatures and more frequently report nicotine burn in the throat. Young smoking individuals may be more likely to respond to the irritant qualities of potentially noxious substances. Lastly, the location of evoked sensory effects of menthol, nicotine and cinnamaldehyde differ within the intra-oral regions.

## Ethics statement

This study was carried out in accordance with the recommendations of GCP, ethics committee of Northern Jutland, with written informed consent from all subjects. All subjects gave written informed consent in accordance with the Declaration of Helsinki. The protocol was approved by the ethics committee of Northern Jutland (case number N-2013-0043).

## Author contributions

SB conceived and designed the study. EH was responsible for data collection and performed statistical analyses. EH and SB interpreted the statistical findings and created the figures. EH drafted the first version of the manuscript. EH, SB, and LA edited, revised, and approved the final manuscript.

### Conflict of interest statement

SB is the co-inventor of the Navigate Pain™ software application used to collect the oral pain drawings and has financial holdings in the company Aglance Solutions. The authors declare that this study received funding from the Nicotine Science Center, Fertin Pharma. The funder also manufactured the gums required for this study. The funder was not involved in the study design or collection, analysis, or interpretation of the data.
