# Supplementary material for: A Comparison of Oral Sensory Effects of Three TRPA1 Agonists in Young Adult Smokers and Non-smokers
Source: Front Physiol. 2017 Sep 7;8:663. doi: 10.3389/fphys.2017.00663 (PMC5594084; doi:10.3389/fphys.2017.00663)
Supplement: Supplementary file 1 [file DataSheet1.PDF]

Supplemental Figure 1:

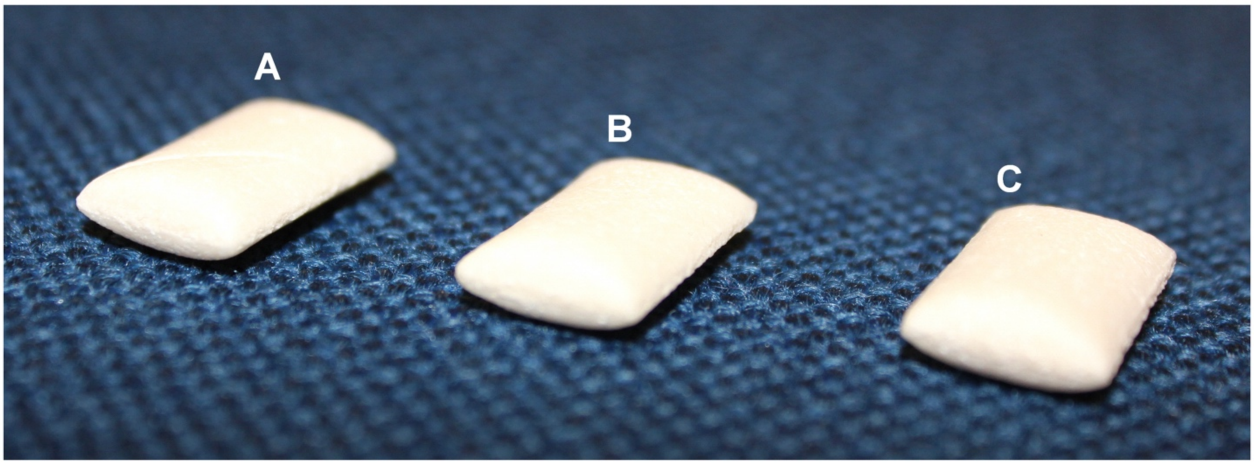

Supplemental Figure 1: A photograph showing the size, color (white), texture and shape of menthol (A), nicotine (B) and cinnamaldehyde (C) gums.

Supplemental Figure 2:

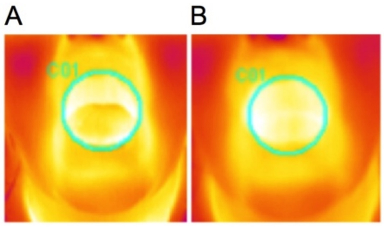

Supplemental Figure 2: Thermographs recorded at baseline (A) and at 5 min (B) of chewing menthol gum. The circle, which borders the corners of the mouth and upper and lowers lips, is the area where mean temperature was calculated and subsequently extracted for each thermograph.

Supplemental Figure 3:

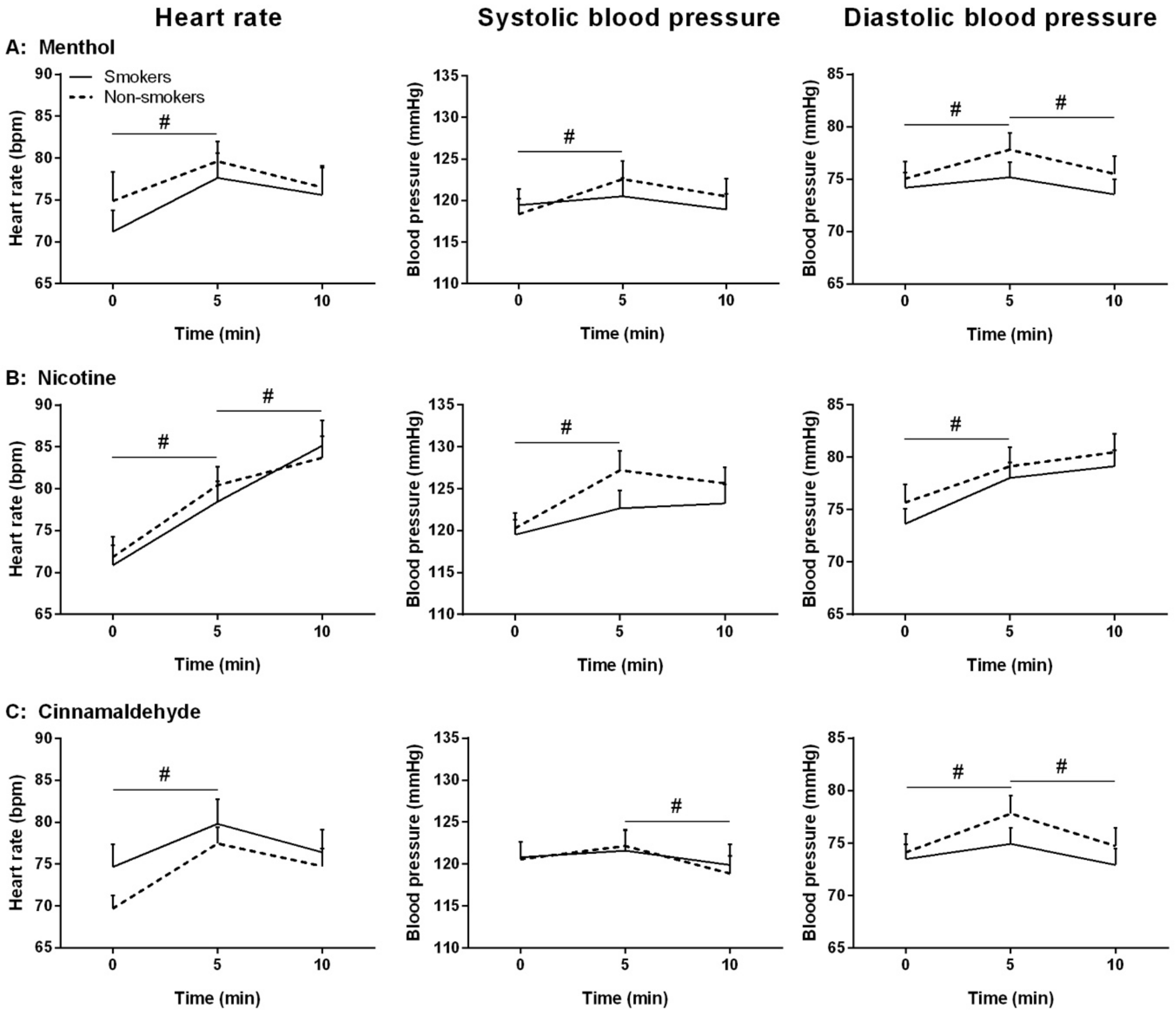

Supplemental Figure 3: Heart rate, systolic and diastolic blood pressure in response to menthol (A), nicotine (B) and cinnamaldehyde (C) before, during (5 min) and after chewing (10 min). Heart rate and blood pressure responses were similar for smokers and non-smokers. #Significant change over time. Data presented as mean ± SEM.

Supplemental Figure 4:

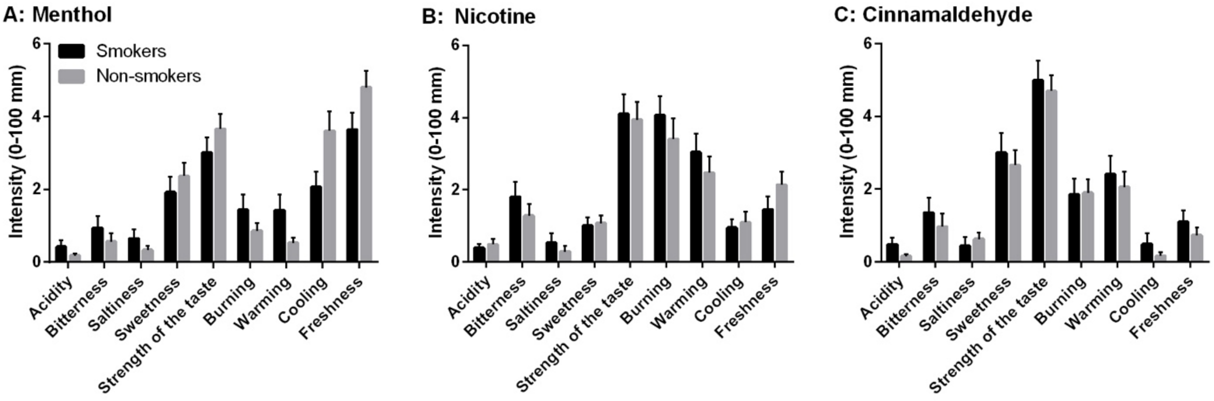

Supplemental Figure 4: Taste experiences in response to menthol (A), nicotine (B) and cinnamaldehyde (C) for smokers and non-smokers. The responses were similar for smokers and non-smokers. Data presented as mean ± SEM.
